# Low-dose paclitaxel inhibits the induction of epidermal-mesenchymal transition in the human cholangiocarcinoma CCKS-1 cell line

**DOI:** 10.3892/ol.2013.1494

**Published:** 2013-07-25

**Authors:** ATSUSHI HIROSE, HIDEHIRO TAJIMA, TETSUO OHTA, TOMOYA TSUKADA, KOICHI OKAMOTO, SHINICHI NAKANUMA, SEISHO SAKAI, JUN KINOSHITA, ISAMU MAKINO, HIROYUKI FURUKAWA, HIRONORI HAYASHI, KEISHI NAKAMURA, KATSUNOBU OYAMA, MASAFUMI INOKUCHI, HISATOSHI NAKAGAWARA, TOMOHARU MIYASHITA, HIROYUKI TAKAMURA, ITASU NINOMIYA, HIROHISA KITAGAWA, SACHIO FUSHIDA, TAKASHI FUJIMURA, SHINICHI HARADA

**Affiliations:** 1 Department of Gastroenterologic Surgery, Division of Cancer Medicine, Kanazawa University, Kanazawa, Ishikawa 920-8640, Japan; 2 Center for Biomedical Reserch, Graduate School of Medicine, Kanazawa University, Kanazawa, Ishikawa 920-8640, Japan

**Keywords:** cholangiocarcinoma, paclitaxel, epidermal-mesenchymal transition

## Abstract

Epidermal-mesenchymal transition (EMT) confers an advantage to cancer cells by improving their invasive capacity and metastatic potential. This phenomenon by which epidermal cells change into mesenchymal cells and therefore acquire a higher ability to automaticity, is considered a key process in cancer development. Transforming growth factor-β (TGF-β) is a significant factor for accelerating EMT through the activation of proteins, including members of the Smad pathway. Furthermore, previous studies have shown that low-dose paclitaxel (PTX) inhibits EMT in certain cell lines, including those of cancer cells. The present study determined whether low-dose PTX was able to inhibit EMT in a human cholangiocarcinoma CCKS-1 cell line that had been treated with TGF-β1. First, the cytotoxic concentration of PTX for the CCKS-1 cells was identified to be ~5 nM by MTT assay and dead cell staining. Therefore, the concentrations of PTX were set as 1 nM, 2.5 nM and 5 nM for the subsequent experiments. In the morphological investigation, the CCKS-1 cells changed into a spindle morphology and became separated by the administration of TGF-β1. However, low-dose PTX inhibited these changes and the morphology resembled the control cells in a dose-dependent manner. Similarly, immunofluorescence and immunoblotting investigations revealed that the CCKS-1 cells expressed mesenchymal markers following the administration of TGF-β1. However, low-dose PTX inhibited the expression of the mesenchymal markers and the CCKS-1 cells expressed the epithelial marker, E-cadherin. In particular, a concentration-dependent effect was observed in the immunoblotting experiments. These results show that PTX may be able to inhibit EMT in cancer cells, depending on the dose concentration.

## Introduction

Cholangiocarcinoma is one of the most lethal malignant tumors (1), as it is difficult to diagnose in the early stages. Since symptoms develop later, patients are often diagnosed when the cancer is at a metastatic stage (2,3). Numerous other malignant tumors are also difficult to diagnose in their early stages. As a result, identifying a method of curative therapy for advanced-stage tumors is urgently required. Invasion and metastasis are significant factors in the advanced stages of tumors. The inhibition of these phenomena may enhance the treatment outcome of malignant tumors and also allow the patient to be treated with a resection or using chemotherapy, thus inhibiting the appearance of new lesions despite being at an advanced stage.

Epidermal-mesenchymal transition (EMT) is a process whereby epidermal cells exhibit reduced intercellular adhesion and acquire fibroblast-like properties (4). This phenomenon is common to normal development and carcinogenesis, and is associated with mechanisms that induce tumor invasion and metastasis (5). By this process, epidermal cells are converted to cells that display mesenchymal features and become dedifferentiated and malignant. Biological markers of EMT include a decrease in the level of epithelial markers, including E-cadherin, and the expression of mesenchymal markers, including N-cadherin, vimentin and α-smooth muscle actin (α-SMA) (6–8). Furthermore, transforming growth factor-β (TGF-β) induces EMT in tumor cells, activating the TGF-β signaling pathway, which includes the Smad proteins and also the non-Smad pathways (9,10).

In contrast, certain studies have demonstrated that paclitaxel (PTX), one of the major anticancer agents that stabilizes microtubules and arrests the cell cycle in the G_0_/G_1_ and G_2_/M phases (11,12), inhibits the invasive ability of breast cancer cell lines when used in low-doses (13,14). Furthermore, certain studies have revealed that low-dose PTX inhibits TGF-β/Smad activity in fibrosis (15,16). Based on these findings, the present study hypothesized that low-dose PTX may inhibit the induction of EMT by TGF-β1 in the human cholangiocarcinoma CCKS-1 cell line.

## Materials and methods

### Reagents

PTX (moleculer weight, 853.91; Santa Cruz Biotechnology, Inc., Santa Cruz, CA, USA) and TGF-β1 (Sigma-Aldrich, St. Louis, MO, USA) were used.

### Antibodies

Mouse monoclonal antibodies for N-cadherin, E-cadherin, α-SMA, β-catenin and vimentin were used as primary antibodies. A rabbit monoclonal antibody was used for N-cadherin and a goat polyclonal antibody for phosphorylated (p)-Smad2/3 (Santa Cruz Biotechnology, Inc.).

### Cell culture

A human ICC cell line, CCKS-1, obtained from the Department of Human Pathology, Kanazawa University Graduate School of Medicine (Kanazawa, Ishikawa, Japan) (17,18) was used. The ICC cell line was maintained at 37°C in a 5% CO_2_ incubator and grown in RPMI-1640 medium supplemented with 2 mM glutamine, 1% fetal bovine serum (FBS; Nichirei Biosciences, Inc., Tokyo, Japan), 100 U/l penicillin and 100 μg/ml streptomycin (Invitrogen, Carlsbad, CA, USA).

### Cell proliferation assay

The proliferative effect of PTX on the ICC cell lines was quantified using an MTT colorimetric assay with Cell Proliferation kit I (Roche, West Sussex, UK), according to the manufacturer’s instructions. In brief, the CCKS-1 cells (5×10^3^ cells/well) were grown in 96-well flat-bottom microtiter plates in 100 μl medium containing 1% FBS and incubated for 12 h at 37°C in a humidified atmosphere with 5% CO_2_. Following the incubation period, the medium was exchanged for a new medium containing 1% FBS, 5 ng/ml TGF-β1 and/or various concentrations (1–100 nM) of PTX. The mixture was incubated for 72 h and the resultant absorbance was recorded at 562 nm using a 96-well plate reader (Multiskan GO; Thermo Scientific, Waltham, MA, USA). The data are represented as the mean ± SD of three independent experiments and expressed as a percentage of the untreated control cells.

### Cell death assay

The cytotoxic effect of PTX on the ICC cell lines was quantified by flow cytometry using Pacific Blue™ annexin V and SYTOX^®^ AADvanced™ dead cell stain (Invitrogen), according to the manufacturer’s instructions. In brief, the CCKS-1 cells were cultured in a medium containing 10% FBS. Following the culture, the medium was exchanged for new medium containing 1% FBS and/or various concentrations of PTX with 5 ng/ml TGF-β1 (control, 5 ng/ml TGF-β1 only; 1 nM PTX + TGF-β1; 2.5 nM PTX + TGF-β1; 5nM PTX + TGF-β1; and 10 nM PTX + TGF-β1) and incubated for 7 days. Following the incubation period, the cells, including the floating cells, were harvested and washed in cold PBS. The cells were resuspended in 1X annexin binding buffer at ~1×10^6^ cells/ml, preparing a sufficient volume to be able to use 100 μl per assay. Subsequently, 5 μl Pacific Blue annexin V and 1 μl 500 μM SYTOX AADvanced dead cell stain working solution were added to each cell suspension and incubated at room temperature for 30 min, protected from the light. Following the incubation period, 400 μl 1X annexin binding buffer was added to each suspension. During the analysis, the samples were kept on ice.

### Investigating the optimal administration interval between PTX and TGF-β

An immunoblotting analysis of p-Smad2/3 was used to investigate the optimal administration interval between PTX and TGF-β. Following the culture period, the medium was replaced with RPMI-1640 medium containing 1% FBS and 2.5 nM PTX. Subsequently, 5 ng/ml TGF-β was administered after PTX using one of the following intervals; 0, 10, 30 and 120 min. Following 60 min of the TGF-β reaction time as described, the cells that did not undergo apoptosis or the dead cells that were floating in the medium were harvested by trypsinization using 0.25% trypsin-EDTA (Invitrogen), then washed 3 times with PBS and dissolved in RIPA buffer (Wako Pure Chemical Industries, Ltd., Osaka, Japan) containing protease and phosphatase inhibitors (Sigma-Aldrich). The protein concentration of each sample was measured using a BCA protein assay kit (Thermo Scientific). The total protein was measured using a spectrophotometer. The extracted protein was used for the western blot analysis. In this analysis, 45 μg protein from each sample was loaded onto 12.5% sodium dodecyl sulfate-polyacrylamide gels (SDS-PAGE) and the proteins were transferred to a polyvinylidene difluoride (PVDF) membrane by the semi-dry blotting method using blotting solution (hydroximethyl; Ez Fast Blot; ATTO Corporation, Tokyo, Japan). The membrane was washed for 10 min with blocking solution (0.1% Tween-20; Ez Block; ATTO Corporation), blocked at room temperature for 30 min again using blocking solution and then washed with washing solution (0.1% Tween-20; Ez Wash; ATTO Corporation). The blots were incubated for 8 h at room temperature with a goat anti-p-Smad2/3 antibody diluted at 1:500 with washing solution. Subsequent to being washed with a gradient buffer (Ez Wash), the membranes were incubated with an HRP-conjugated anti-goat IgG antibody for 1 h at room temperature. The antibody-antigen complex was detected using the ECL Plus Western blotting detection system (GE Healthcare UK, Ltd., Buckinghamshire, UK), according to the supplier’s recommendations.

### Immunocytochemistry

The expression of E-cadherin, N-cadherin, vimentin and β-catenin in the ICC cells was examined immunocytochemically using their respective primary antibodies. The cells were seeded on Lab-Tek chamber slides (Nalge Nunc International, Penfield, NY, USA) with PTX and/or TGF-β (5 ng/ml TGF-β; 1 nM PTX + 5 ng/ml TGF-β; 2.5 nM PTX + 5ng/ml TGF-β; or 5 nM PTX + 5 ng/ml TGF-β) and incubated for 7 days at 37°C in a humid atmosphere of 5% CO_2_/95% air. Following the incubation period, the waste solution was discarded and the coverslips with the cells were then fixed with methanol and acetone 1:1 (v/v) for 10 min.

Immunostaining was performed as described. Briefly, the slides were blocked with normal goat serum [5% in phosphate-buffered saline (PBS)] and incubated with each primary mouse monoclonal antibody, as described previously, for 6 h at room temperature. The slides were washed in PBS and the immunoreactivity was visualized by incubating the slides with a goat anti-mouse IgG antibody conjugated with Alexa Fluor 488 (Invitrogen; 1:400) for 1 h at room temperature. The slides were counterstained with bis-benzimide (100 ng/ml; Hoechst 33258; Sigma-Aldrich) to visualize the nuclei. The slides were then examined under a fluorescence microscope (BZ-9000 Biorevo; Keyence, Osaka, Japan). In addition, the cadherin switch was examined by double staining. A primary rabbit monoclonal antibody against N-cadherin was administered and incubated for 6 h at room temperature following incubation with the primary mouse monoclonal antibody against E-cadherin and being washed in PBS. A goat anti-mouse and anti-rabbit IgG antibody were used at the same time.

### Immunoblot analysis

The processes for harvesting and measuring the protein concentration and for the blotting technique were the same as described previously. Briefly, each sample used 45 μg protein and the 4 antibodies for E-cadherin (1:100), N-cadherin (1:100), vimentin (1:500) and α-SMA (1:500) in the western blot analysis. The antibodies were used as EMT markers to measure the up or downregulation of the expression in the CCKS-1 cells that were incubated in the medium with an added concentration of PTX and/or TGF-β1 (control, 5 ng/ml TGF-β1; 1 nM PTX + 5 ng/ml TGF-β1; 2.5 nM PTX + 5 ng/ml TGF-β1; and 5 nM PTX + 5ng/ml TGF-β1) for 8 h at room temperature.

## Results

### Determining the optimal PTX concentration

**Figure 1:**
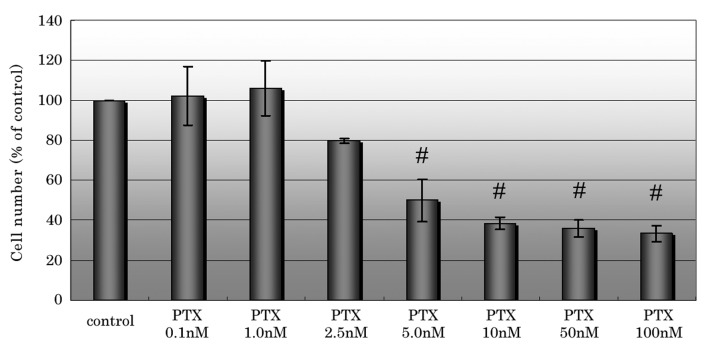
Treatment of CCKS-1 cells with various concentrations of PTX. PTX was observed to start inhibiting cell proliferation at concentrations of ≥2.5 nM and significantly inhibit cell proliferation at concentrations of ≥5 nM. The data are represented as the mean ± SD of three independent experiments. Statistically significant differences were determined by Welch’s t-test. ^#^P<0.01 vs. control. PTX, paclitaxel.

**Figure 2:**
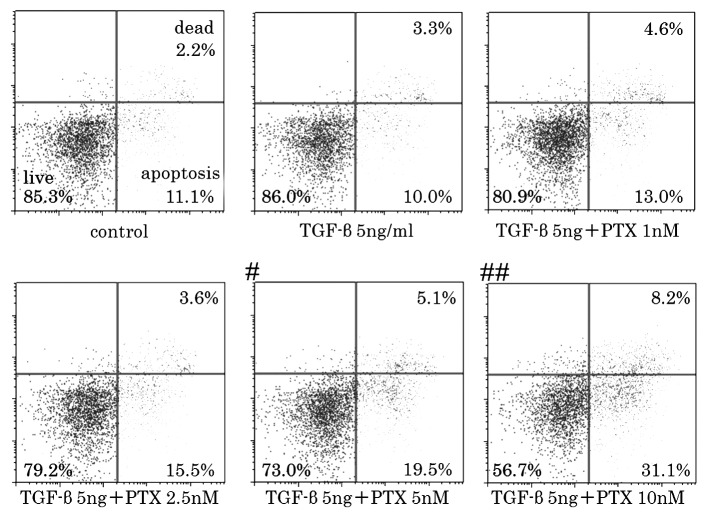
Flow cytometry of CCKS-1 cell death assay using various concentrations of PTX and/or TGF-β. The percentage of apoptotic/dead cells increased at concentrations of ≥5nM and were significantly increased at ≥10nM. Statistically significant differences were determined by the χ^2^ test. ^#^P<0.05; ^##^P<0.01 vs. control. TGF-β1, transforming growth factor-β1; PTX, paclitaxel.

In the cell proliferation assay, PTX inhibited cell proliferation at concentrations of ≥2.5 nM and significantly inhibited cell proliferation at concentrations of ≥5 nM compared with the control (Fig 1). The percentage of the dead and apoptotic cells markedly increased when using a concentration of 5 nM or higher in the cell death assay (Fig. 2). Based on these results, the cytotoxic concentration of PTX for CCKS-1 was estimated to be 2.5–5nM. Therefore, the concentrations that were used in the subsequent experiments were 1, 2.5 and 5 nM PTX. A concentration of 5 nM PTX was included in order to observe whether EMT is inhibited at a cytotoxic concentration.

### Morphological investigation

**Figure 3:**
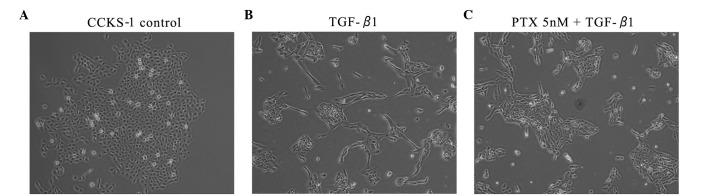
Morphological investigations of CCKS-1. (A) Untreated CCKS-1 cells showing a pebble-like shape with cell-cell contact. (B) CCKS-1 cells became spindle-shaped and the cell-cell contacts separated (5ng/ml TGF-β1). (C) Low-dose PTX lead to close cell-cell contacts and pebble-like shaped cells in contrast with the TGF-β1-treated cells (5ng/ml TGF-β1 + 5 nM PTX). TGF-β1, transforming growth factor-β1; PTX, paclitaxel.

The untreated CCKS-1 cells displayed a cobblestone-like morphology and cell-to-cell adhesion was intact. However, the TGF-β1-treated CCKS-1 cells developed a spindle-shaped morphology, the cell-to-cell adhesions became weak and the cells were scattered. The cells that were treated with low-dose PTX were assembled closely, although the morphology did not completely resemble the cobblestone-like appearance of the control cells. The morphological changes were concentration-dependent (Fig. 3).

### Immunofluorescence investigation

**Figure 4:**
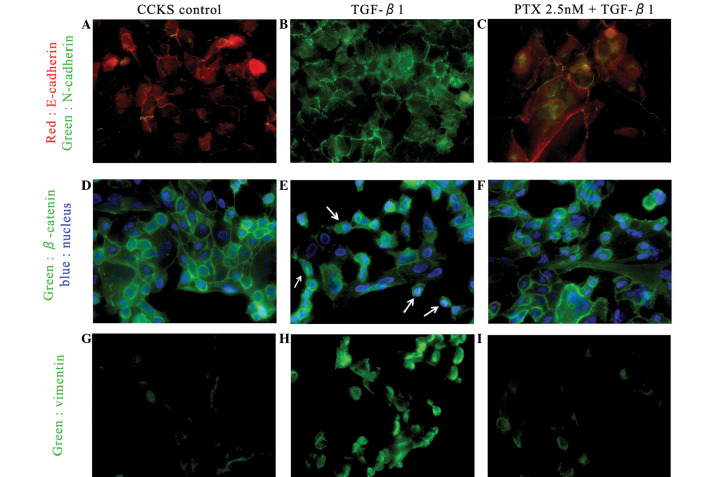
Immunofluorescence investigations of the cadherin switch (red, E-cadherin; green, N-cadherin). (A) Untreated CCKS-1 cells strongly express E-cadherin on the cell membrane, but the administration of 5ng/ml TGF-β1 leads to the cadherin switch. (B) CCKS-1 cells strongly express N-cadherin. (C) CCKS-1 cells treated with low-dose PTX express E-cadherin more strongly than N-cadherin (5ng/ml TGF-β1 + 2.5nM PTX). Immunofluorescence investigations of β-catenin (green, β-catenin; blue, nuclei). (D) Untreated CCKS-1 cells express β-catenin on the cell membrane and cytoplasm. (E) TGF-β1-treated CCKS-1 cells express β-catenin in the nucleus (arrow; 5ng/ml TGF-β1), but (F) low-dose PTX inhibited those changes (5 ng/ml TGF-β1 + 2.5nM PTX). Immunofluorescence investigations of vimentin. (G) Untreated CCKS-1 cells weakly express vimentin, but (H) TGF-β1-treated CCKS-1 cells express vimentin strongly (5ng/ml TGF-β1). (I) Low-dose PTX inhibits the expression of vimentin, similar to the untreated cells (5ng/ml TGF-β1 + 5 nM PTX). TGF-β1, transforming growth factor-β1; PTX, paclitaxel.

In the investigation of the cadherin switch and vimentin expression, the untreated CCKS-1 cells predominantly expressed E-cadherin on the cell membrane and weakly expressed vimentin. The TGF-β1-treated CCKS-1 cells expressed N-cadherin and vimentin strongly. However, the expression of E-cadherin in the low-dose PTX-treated CCKS-1 cells was stronger than the expression of N-cadherin, and the vimentin expression was weak, which was consistent with the control. Similarly, the untreated CCKS-1 cells expressed β-catenin on the cell membrane and in the cytoplasm. In the TGF-β1-treated CCKS-1 cells, β-catenin expression shifted partially to the nucleus (arrows), but the administration of low-dose PTX inhibited this movement (Fig. 4).

### Immunoblot investigation

**Figure 5:**
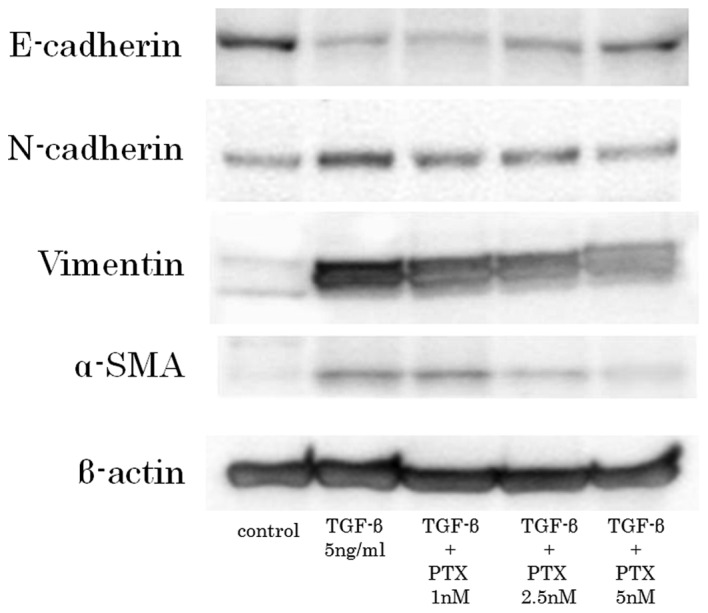
Western blot analysis of epidermal/mesenchymal markers. Untreated CCKS-1 cells weakly express the mesenchymal markers. However, TGF-β1-treated CCKS-1 cells strongly express the mesenchymal markers. Low-dose PTX inhibits mesenchymal change in a concentration-dependent manner. TGF-β1, transforming growth factor-β1; PTX, paclitaxel; α-SMA, α-smooth muscle actin.

The untreated CCKS-1 cells strongly expressed E-cadherin and weakly expressed the mesenchymal markers. In contrast, the TGF-β1-treated CCKS-1 cells expressed the mesenchymal markers strongly and E-cadherin weakly. However, the low-dose PTX weakened the expression of the mesenchymal markers and enhanced E-cadherin expression in a concentration dependent manner (Fig. 5).

## Discussion

EMT is a concept by Hay *et al* that is observed during the mesenchymal transition of primitive epidermal cells during gastrulation (19). In subsequent studies, EMT has been classified into three subtypes (20). Type 1 EMT is involved during developmental stages, including gastrulation, the migration of neural crest cells from neuroepithelial cells and the formation of endocardial cushion tissue from cardiac endothelial cells. Type 2 EMT involves the transition of epidermal cells to tissue fibroblasts, which participate in wound healing, regeneration and fibrosis in adult tissues. Type 3 EMT involves the metastatic or invasive process of carcinoma. However, while these three processes are different, there are certain aspects in common in the induction of the EMT mechanism. TGF-β is a well-known factor to induce EMT (9,10). Although the detailed explanation with regard to the association between TGF-β signaling and EMT is skipped, the Smad pathway is significant to the signaling process. In brief, p-Smad2/3 forms heteromeric complexes with Smad4, which translocate into the nucleus and act as transcriptional regulators of target genes by interacting with other transcription factors and transcriptional regulators.

Recently, several anti-EMT agents have been reported in *in vitro* analyses, including vorinostat (21), panobinostat (22), valproic acid (23) and PTX. As discussed previously, low-dose PTX has been shown to inhibit fibrosis and the invasive ability of cancer cells in several cell lines. A low dose of PTX is considered to suppress the phosphorylation of Smad2/3. Although it is well known that PTX behaves as an anticancer agent by stabilizing microtubules, it is also been identified that microtubules and Smad 2/3 are closely connected (24). Taken together, this data indicates that PTX acquires anticancer abilities by regulating Smad 2/3, which is closely connected with the progression of all types of EMT. PTX, through the regulation of Smad 2/3, has the potential to inhibit fibrosis and the invasive abilities of cancer.

The present study was performed based on these aforementioned phenomena. PTX was observed to potentially inhibit EMT in CCKS-1 cells, as shown by the aspects of their morphology and the inhibition of the expression of mesenchymal markers on immunofluorescence and immunoblot investigations. However, it is noteworthy that PTX inhibited the expression of mesenchymal markers in a concentration-dependent manner in the immunoblotting investigation. The optimal PTX concentration was estimated at 5 nM PTX, as this was the cytotoxic concentration for the CCKS-1 cells. Accordingly, PTX may potentially inhibit EMT in low-dose and normal-dose concentrations for viable CCKS-1 cells. Using 10 nM concentrations of PTX to examine this is difficult, since few cells are able to survive in strongly cytotoxic concentrations, therefore, further study is required. Furthermore, the present study also indicates further investigation is warranted into the role of the pathways that include Smad2/3 in EMT.

As biliary tract cancers, including cholangiocarcinoma, are difficult to diagnose in their early stage, there are numerous cases that are treated with chemotherapy. While one of the major chemotherapeutic regimens for biliary tract cancers is cisplatin with gemcitabine, as established by Valle *et al*(25), the median overall survival time is only 11.7 months. Other regimens based on 5-fluorouracil (FU), one of the major drugs that is used in the treatment of hepatobiliary-pancreatic cancers, were not shown to contribute to survival and quality of life (26,27).

Furthermore, it has been reported that anti-cancer treatments, including chemotherapy, radiation therapy and radiofrequency ablation, may induce EMT in cancer cells (28–30). Based on these studies, major anticancer treatments using anti-EMT treatments, including low-dose PTX, may be truly effective treatment approaches. In a previous study, we experienced a case of successful treatment for unresectable gallbradder cancer with low-dose PTX following the failure of gemcitabine and oral S-1 (31), which is an oral prodrug for 5-FU that is widely used in Japan (32).

In conclusion, though the possibility of inhibiting EMT using PTX for biliary tract cancers in clinical practice is unclear, the potential of PTX warrants further investigation.
